# Extraction Protocol for Parallel Analysis of Proteins and DNA from Ancient Teeth and Dental Calculus

**DOI:** 10.1021/acs.jproteome.3c00370

**Published:** 2023-09-12

**Authors:** Eva Chocholova, Pavel Roudnicky, David Potesil, Dana Fialova, Karolina Krystofova, Eva Drozdova, Zbynek Zdrahal

**Affiliations:** † Laboratory of Biological and Molecular Anthropology, Department of Experimental Biology, Faculty of Science, Masaryk University, Kamenice 5, 62500 Brno, Czech Republic; ‡ Mendel Centre for Plant Genomics and Proteomics, Central European Institute of Technology, Masaryk University, Kamenice 5, 62500 Brno, Czech Republic; § National Centre for Biomolecular Research, Faculty of Science, Masaryk University, Kamenice 5, 62500 Brno, Czech Republic

**Keywords:** bioarcheology, paleoproteomics, ancient DNA, ancient proteins, dental calculus

## Abstract

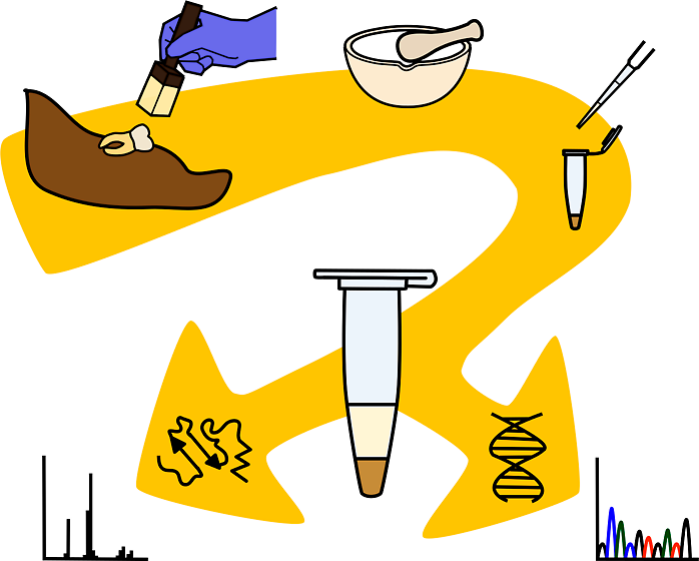

Dental calculus is becoming a crucial material in the study of past populations with increasing interest in its proteomic and genomic content. Here, we suggest further development of a protocol for analysis of ancient proteins and a combined approach for subsequent ancient DNA extraction. We tested the protocol on recent teeth, and the optimized protocol was applied to ancient tooth to limit the destruction of calculus as it is a precious and irreplaceable source of dietary, microbiological, and ecological information in the archeological context. Finally, the applicability of the protocol was demonstrated on samples of the ancient calculus.

## Introduction

The field of paleoproteomics is undergoing rapid development, especially with the growing focus on dental calculus since the discovery of milk proteins inside.^1^ The greatest advantage of ancient protein research is that proteins are better preserved compared to DNA and can survive for millions of years,^2–4^ especially when they bind to mineral surfaces such as those found in teeth or mineralized dental calculus.

Dental calculus represents a valuable source of information. It carries various particles that enter the oral cavity, allowing us to study the composition of the diet,^1,5–12^ oral microbiome,^13–18^ immune reaction,^17^ mitochondrial haplogroups,^19,20^ or even the occupation and habits of an individual.^21,22^ The discovery of ancient DNA (aDNA) and proteins in calculus revolutionized biomolecular archeology and brought a fresh new perspective to the research of past populations, enabling the acquisition of direct evidence for some of the abovementioned behaviors for the first time.

The process of the deposition of dental plaque depends on many variables that are not yet fully understood. Many factors such as pH, oral microbiome, amount, or even chance determine whether a certain piece of debris is caught and later preserved in a calcified plaque–calculus (reviewed by, e.g., Jin *et* Yip^23^). Thus, the layers and segments of the calculus have a variable composition of bacteria, food residues, host biomolecules, or environmental particles. In this way, the dental calculus represents samples with high heterogeneity.

The current objective is to find the most appropriate methodological approach for the extraction and analysis of ancient proteins together with guidelines for the authentication of the results.^9,24–28^ However, optimization of molecular methods on ancient samples is problematic as this material is unique and irreplaceable and needs to be regarded as such. Testing on ancient samples significantly reduces the possibility of using an adequate number of replicates and robusticity of optimizations as they are scarce. Consequently, most studies do not describe whether and how the used method was tested, and the frequently adopted solution is to use the previously applied protocol without any or only minor modifications. Thus, the utilization of recent samples for optimization of sample preparation protocols prior to mass spectrometric analysis could be a solution to limit the destruction of precious ancient material and allow proper testing of the method performance, as was done by, e.g., Schroeter et al.^29^

Technically, methods of protein extraction that do not completely degrade the material are preferred. Acid etching used for sex determination from enamel is less destructive and is useful for this narrow research question.^30–34^ However, such methods might not be efficient enough and do not allow complete information to be obtained on the protein content of complex samples. The development and testing of methods for complex samples, such as calculus, is always needed, and it is suggested that they need to be carefully tested.^35^

One of the key steps of the sample preparation procedure is considered to be demineralization as the subgingival calculus is composed of about 58% of the mineral component, the supragingival one has 37%,^36^ while dentin is mineralized from 70% and dental enamel from 97%.^37^ The mineralized portion increases with the process of fossilization and decay of organic matter, which brings the mineral composition of ancient samples even closer to that of dentin and enamel.

Most studies use EDTA overnight for up to 7 days for demineralization.^5,9,35,38,39^ In some protocols, SDS is added for the decalcification step.^9,10^ Further along, usual preparation is based on the filter aided sample preparation (FASP) protocol published by Wiśniewski et al.^40^—usually modified for smaller volumes and ancient samples by Jeong et al.^38^ and Cappellini et al.^41^ (used further by, e.g., Bleasdale et al.,^5^ Charlton et al.,^6^ Fagernäs et al.,^35^ Geber et al.,^7^ and Scott et al.^39^), or gel-aided sample preparation (GASP).^9,10^ In this study, we performed a comparison of several protocols for protein extraction using recent teeth to limit the destruction of ancient material and proposed a combined protocol that allows protein and DNA isolation from the same tooth or calculus material, adopting a different approach to the current protocol for unified decalcification^35^ to offer an alternative for processing of ancient calculus samples. The proposed protocol was applied to ancient teeth and calculus.

## Materials and Methods

### Experiment Design

**Figure 1:**
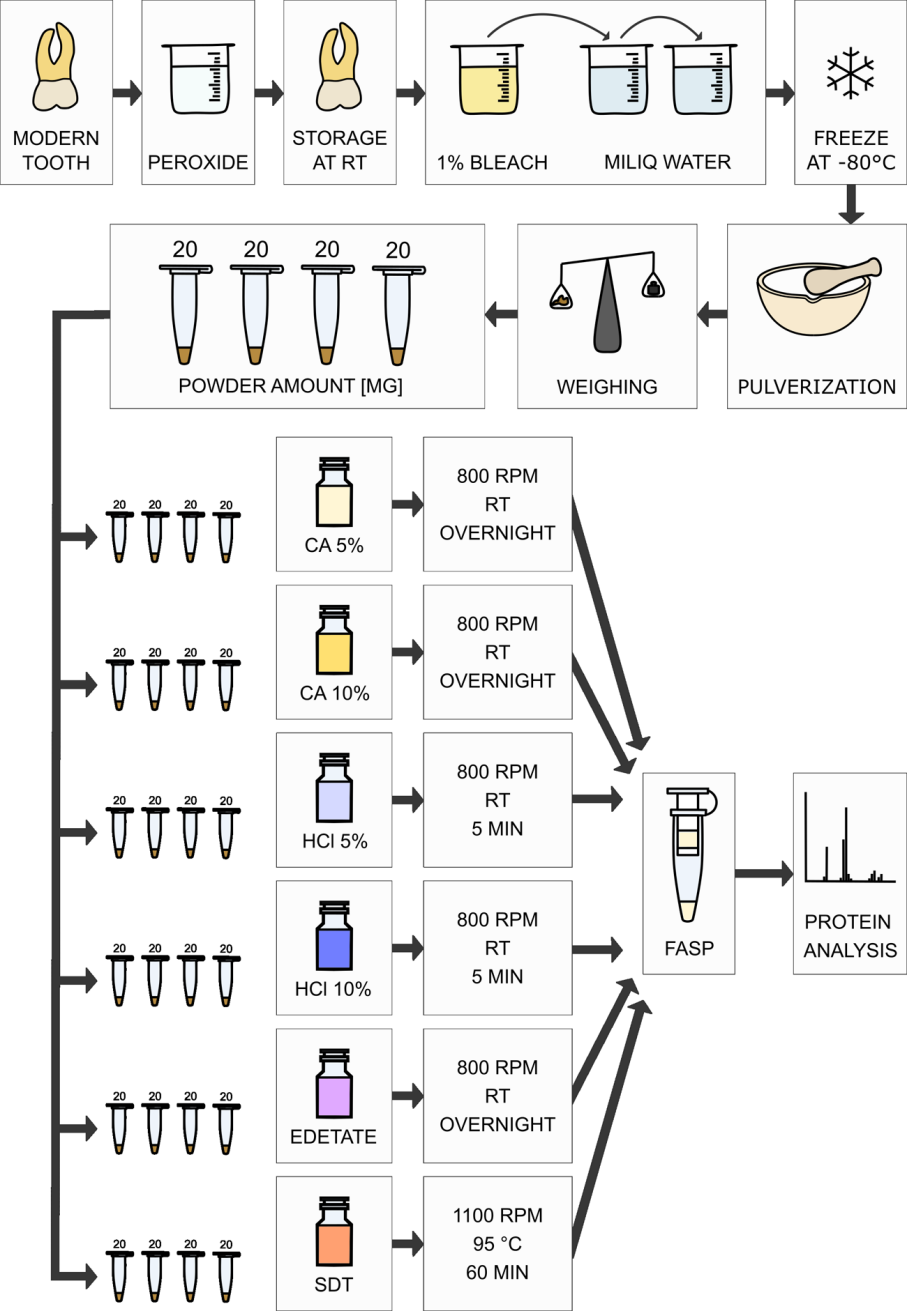
Laboratory workflow of protein analysis of modern teeth. Decontamination, freezing, and pulverization were used for preparation of pooled modern tooth powder. Next, four replicates were prepared from this pool for each tested treatment. Input material of each replicate was about 20 mg.

**Figure 2:**
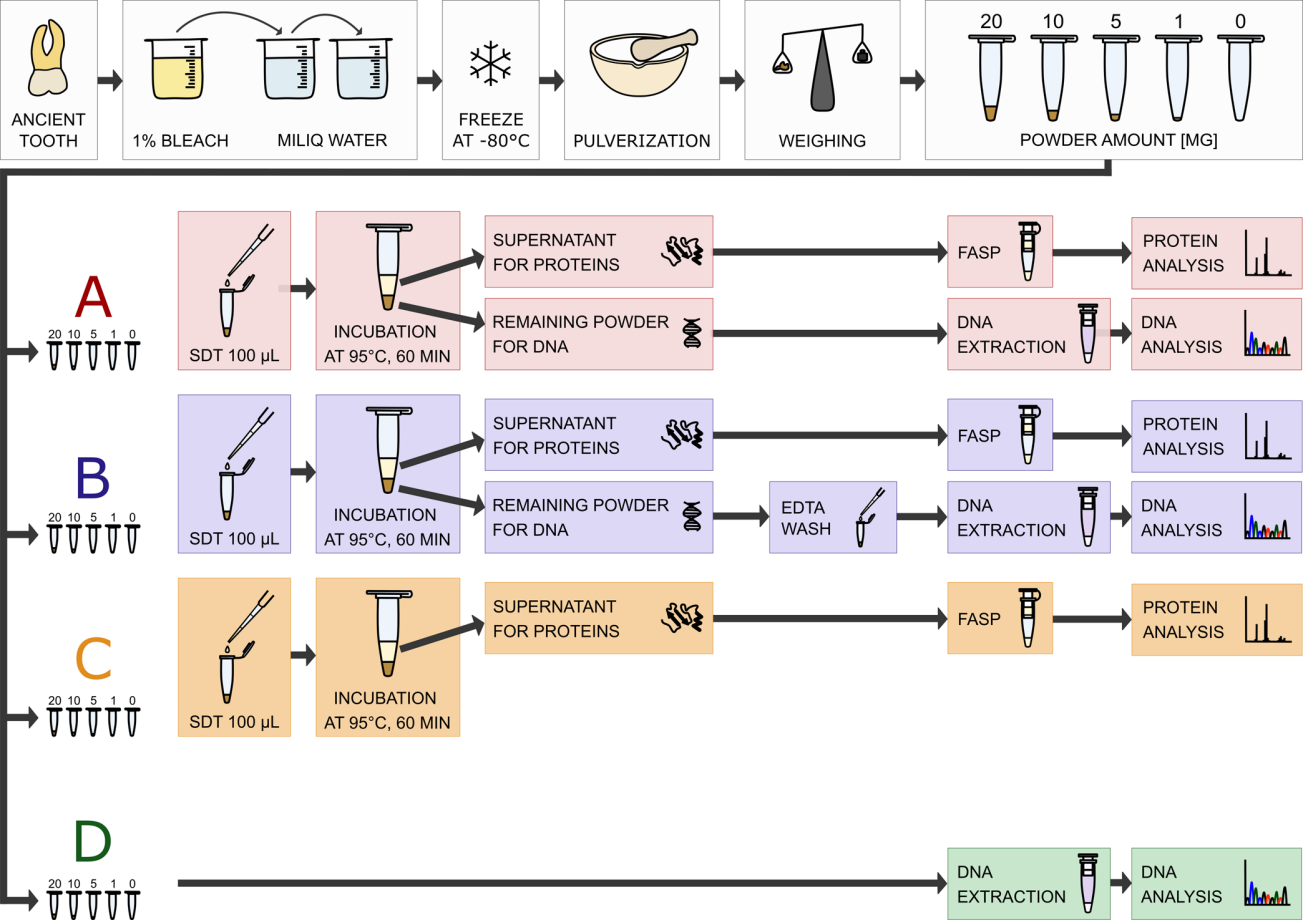
Laboratory workflow of protein and DNA analyses of the ancient tooth. After decontamination, freezing, and pulverization, the obtained tooth powder was divided into four sample groups by weighing. Each group consisted of four samples differing in input material (∼20, 10, 5, and 1 mg) plus a blank (0 mg, extraction control). The samples in the A and B groups were analyzed for both protein and DNA content. A step of EDTA wash prior to DNA extraction was added for B group samples. C group samples were analyzed only by the proteomic approach, and D group samples were utilized only for ancient DNA analysis.

We used teeth from recent patients obtained within the past decade by a dentist to test different extraction conditions on nonancient specimens (see the scheme in Figure 1). To test the efficiency of ancient samples, we applied the best-performing extraction conditions to different weights of the pulverized ancient tooth and confirmed the presence of aDNA in these samples (Figure 2). After confirming the applicability to both recent and ancient teeth, we applied the same methodology to the more precious ancient dental calculus.

### Samples

The origin and dating of the analyzed samples are summarized in Table 1. Two recent upper right third molars (n. 18 according to FDI, World Dental Federation) of males were used for optimization. Further tests were performed on an upper right second molar (n. 17) of a female found at an early medieval site in Znojmo-Hradiště, Czech Republic.^42^ The dental calculus originated from a male from the same burial site (lingual side of tooth n. 35) and two individuals buried in a hospital cemetery dated between the 13th and 16th centuries in Náměstí Svobody, Znojmo, Czech Republic.^43^

**Table 1:** Summary of Samples Used for Protocol Optimization^a^

grave/individual number	sample number	material	location	dating
REC1 and REC2	REC	tooth	18	recent
ZH 539a	ZH539T	tooth	17	early middle ages^b^
ZH 576	ZH576C	calculus	35 lingual	early middle ages^b^
NSZ 151	NSZ151C	calculus	28 mesial	medieval—early modern^c^
NSZ 120	NSZ120C	calculus	11/12 labial	medieval—early modern^c^

a The location of the tooth/calculus is based on the terminology of FDI—World Dental Federation. All archeological sites are located in the Czech Republic, central Europe. The dating was done based on the archeological context of these vast burial sites. Recent teeth REC1 and REC2 were combined to obtain a sufficient amount of tooth powder.

b Znojmo-Hradiště (9th-11th century).

c Náměstí Svobody, Znojmo (13th-16th century).

Recent tooth samples were submerged in 3% hydrogen peroxide for sterilization and stored at room temperature prior to the experiment. Prior to protein extraction, all tooth samples were cleaned with 1% bleach and ultrapure water, frozen at −80 °C for 48 h, and pulverized in an oscillatory mill (Retsch MM 301), see Figure 1.

### Protein Extraction of Recent Samples

For the selection of the best protein extraction approach, ∼20 mg samples of pooled powder from recent teeth REC1 and REC2 (see Table 1, exact amounts in Table S1) were treated with 100 μL of HCl—5 and 10% in MQ water (v/v), citric acid (CA)—5 and 10% in MQ water (w/v), solution of sodium calcium edetate, and SDT buffer (4% SDS, 0.1 M DTT, 0.1 M Tris/HCl, pH 7.6), as shown in Figure 1. Each treatment was applied to four samples that were treated as replicates. Here, we followed common protocols used standardly on modern samples for each extraction agent. After incubation, the samples were centrifuged (10 min, 16,000*g*), and the supernatants were collected.

### Protein Extraction of Ancient Samples

For evaluation of the performance of the selected extraction method in ancient samples, medieval tooth and dental calculi were used (see Table 1, exact amounts in Table S2). In this case, the SDT buffer extraction procedure described above was applied.

First, a single ancient tooth was pulverized and the obtained powder was divided into four groups (A, B, C, and D; see Figure 2). Each group consisted of four tooth samples with input material of about 20, 10, 5, and 1 mg and a blank (extraction control). Identical proteomic analysis was applied to samples from groups A, B, and C, effectively making them technical replicates. Group C samples were analyzed only by proteomic approach, and group D samples were only targeted for aDNA analysis.

Finally, we analyzed three individual samples of dental calculus (see Table 1). These samples could not be analyzed in a technical triplicate due to the low amount of material available. Along with the samples, swabs from the surface of the skeleton and box of ZH 576, and extraction controls (blanks) were processed.

### LC–MS/MS Analyses

Protein extracts were subjected to filter-aided sample preparation as described elsewhere.^40^ The resulting peptides from recent teeth were analyzed by liquid chromatography–tandem mass spectrometry (LC–MS/MS) performed using an UltiMate 3000 RSLCnano system connected to an Orbitrap Fusion Lumos Tribrid spectrometer (Thermo Fisher Scientific). Peptides from the ancient tooth and dental calculus were analyzed on a nanoElute system online coupled with a timsTOF Pro spectrometer (Bruker). See the Supporting Information file for full details regarding the analyses and data evaluation, including the custom database described in Table S3.

### Confirmation of the Presence of Ancient DNA

To confirm the feasibility of subsequent DNA extraction, the protocol was tested on 20, 10, 5, and 1 mg of tooth powder from ZH 539a as previously used for protein extraction (exact amounts are given in Table S7). 100 μL of SDT buffer was added to the powder in the samples of groups A and B (see Figure 2) and processed as described previously. After centrifugation, the supernatant was separated from the pellet. The supernatant was used for protein extraction. The pellet was washed in EDTA in samples of group B, no wash step was added for group A samples.

500 μL aliquot of lysis buffer was added to each tube. The lysis buffer was based on Svensson et al.^44^ and Juras et al.^45^ It consisted of 25 μL of urea (8 M), 25 μL of proteinase K solution (20 mg/mL, Qiagen), and 450 μL of EDTA (0.5 M, pH 8.0, Sigma-Aldrich). For comparison, samples (sample group D in Figure 2) that were not treated with SDT buffer were processed using the standard DNA extraction protocol.

Lysis was performed overnight at 56 °C and 900 rpm, and 450 μL of the supernatant was used for DNA isolation by silica columns according to the protocol by Yang et al.^46^ and Anderung et al.,^47^ using a MinElute PCR Purification Kit (Qiagen) with an elution volume of 60 μL. Concentration was measured with a Qubit dsDNA HS Assay Kit in Qubit 2.0 with 10 μL of isolate, and the samples with the highest and lowest input (1A, 1B, 1D, 4A, 4B, and 4D) were measured on a Fragment Analyzer using a DNF-474 High-Sensitivity NGS Fragment Analysis Kit (Advanced Analytical).

The extracted aDNA and the blanks were amplified using primers for HVI (IFb-16128 and IR-16348) and HVII (IIFa-45 and IIR-287) of mtDNA, as done in Nilsson et al.,^48^ and further sequenced by Sanger sequencing, resulting sequences were run through mtDNAprofiler^49^ and MITOMASTER.^50^ Results were compared to the whole mitogenome previously obtained by Illumina sequencing after target enrichment according to the protocol by Senovska et al.^51^ The whole mitogenome was also evaluated by DamageProfiler^52^ to confirm the authenticity and typical ancient DNA damage.

## Results and Discussion

Initially, we sought for the material to be as homogeneous as possible in a sufficient amount, which allowed us to do reasonable testing and subsequent optimization of the sample preparation methods. We selected a homogenized pooled sample of whole recent teeth based on the assumption that if the extraction protocol shows satisfactory efficiency in protein isolation from highly mineralized samples, less mineralized calculus should not pose a problem. Our initial proposition was supported by the fact that ancient DNA is often extracted from dental calculus by similar methods used for bone and tooth samples, and thus parallel DNA extraction could be involved in our optimized protocol.

A sample of a pulverized tooth provides enough material for experiment replication under various conditions and is more frequent even in the historical context.

Next, the optimized protocol was applied to ancient samples, early medieval tooth, and finally dental calculus, the targeted sample type.

### Protein Extraction

On the basis of reported studies and our experience, we selected several extraction solutions to test the efficiency of the protein extraction step using samples of pulverized recent teeth. We applied solutions of citric acid (5 and 10%), hydrochloric acid (5 and 10%), sodium calcium edetate, which is used for partial tooth demineralization in dentistry, and SDT buffer used for protein lysis during FASP sample preparation of protein samples.^40^

**Figure 3:**
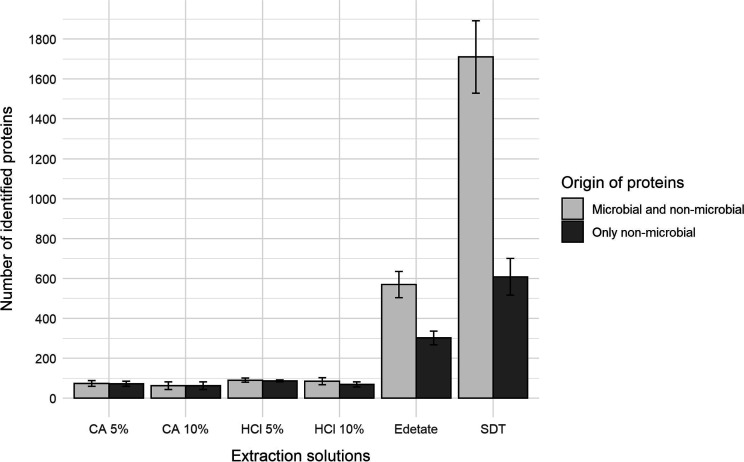
Comparison of the results achieved using different extraction buffers. All measurements were made in quadruplicates. For HCl 5%, one replicate was excluded from the evaluation. The pooled powder from recent teeth was used here (REC1 and REC2). Only proteins identified with at least two unique peptides were considered after filtering the blanks, as described in the Supporting Information file.

Under the given conditions, the highest number of proteins identified by LC–MS/MS analysis was obtained from samples treated with SDT buffer, under elevated temperature (Figure 3, and Table S4). Full results are listed in Datasheet 1. On average, we identified 608 nonbacterial proteins (1710 including microbial ones) compared to less than 100 using acidic treatments. Treatment with edetate allowed for the identification of 302 proteins (570 including microbial ones), providing better results than acidic extraction but still substantially lower than SDT extraction. Taking into account the results, we continued with the application of a SDT buffer for protein extraction in subsequent experiments.

#### Ancient Tooth

**Figure 4:**
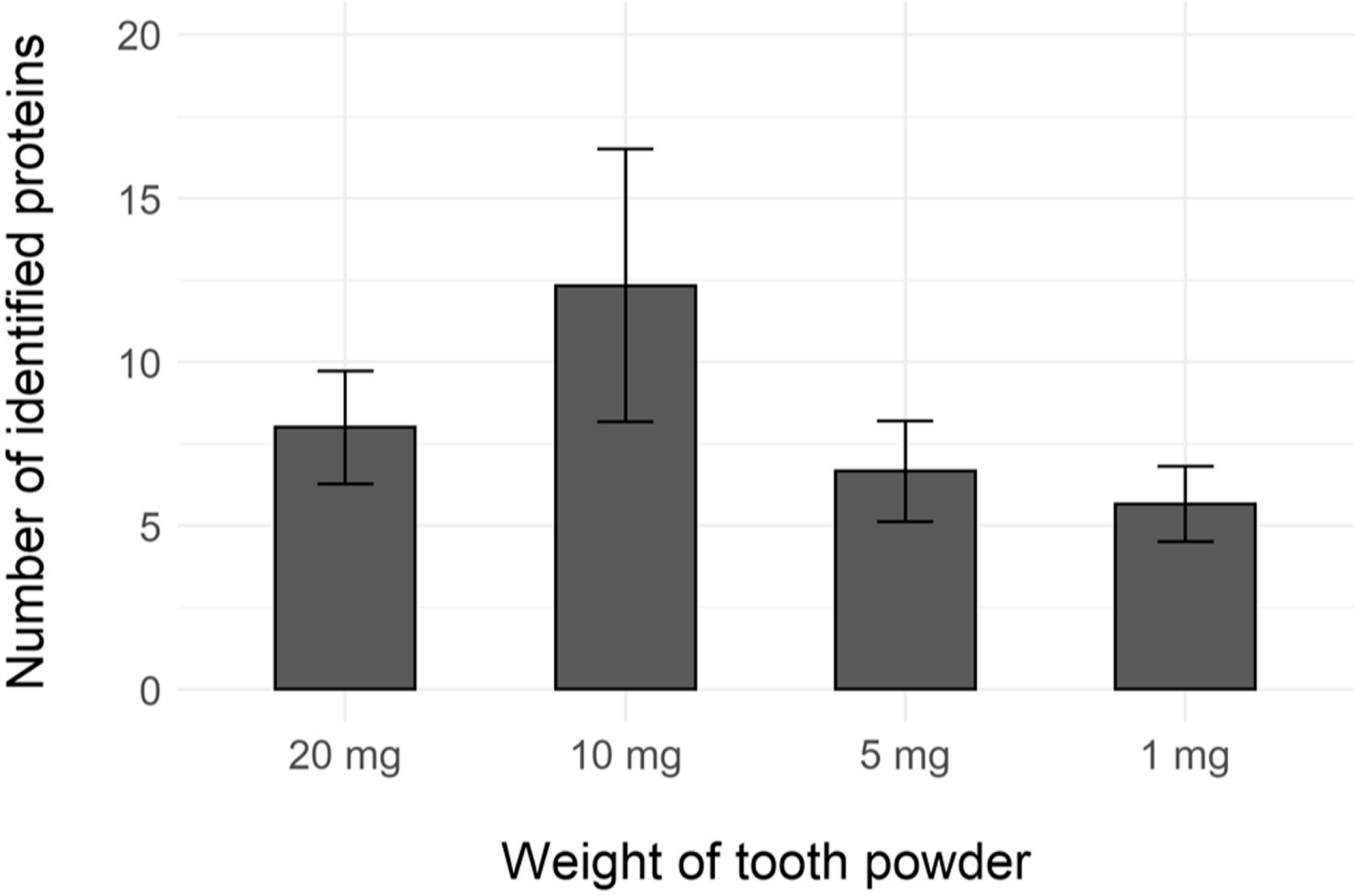
Comparison of the number of identified proteins in different input weights of pulverized ancient tooth. All proteomic measurements were made in triplicates. Only proteins identified with at least two unique peptides were considered after filtering the blanks as described in the Supporting Information.

Next, we applied a selected protocol that combined SDT protein extraction with FASP to an early medieval tooth (ZH539T) as an example of an ancient tooth. After pulverization, we prepared four samples of tooth powder of different weights (1, 5, 10, and 20 mg), each weight in three replicates (sample groups A, B, and C, see Figure 2) for a total of 12 tooth samples and 3 blanks for proteomic analysis. On average we identified 5 to 17 protein groups (PGs) based on 2 unique peptides (12–40 PGs with ≥1 unique peptide) after filtering, with no dependence on the amount of input material. We observed a relatively high variation in the number of identified proteins among individual replicates, which is probably caused by the nonideal homogenization of tooth powder after pulverization (next to the technical variability of the procedure, see Figure 4, and Table S5). Full results are listed in Datasheet 2.

The reduced number of identified proteins (compared to the analysis of recent teeth) is probably a result of native degradation during the time since burial.

#### Ancient Dental Calculus

**Figure 5:**
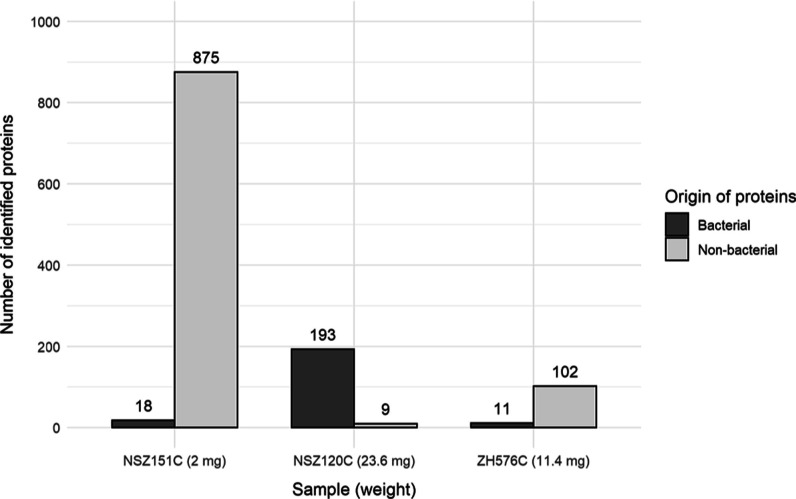
Number of proteins identified in the samples of the ancient dental calculus. The total number of identified proteins is shown, divided into bacterial origin (HOMD) and other (mostly human and probable food). Only proteins identified with at least two unique peptides were considered after filtering the blanks as described in the Supporting Information.

After method optimization using tooth samples, we applied this protocol to three samples of ancient dental calculus (NSZ151C, NSZ120C, and ZH576C). We identified 113–893 proteins based on at least 2 unique peptides (323–1944 with ≥1 unique peptide; Figure 5), including a variety of bacterial, human, and other proteins. The number of identified proteins did not correlate with the amount of sample analyzed, which is consistent with our results (Figure 4) and previous studies.^9,10,25^ Full results are listed in Datasheet 3.

The calculus of individual NSZ 151 (NSZ151C) is composed of a great number of human proteins, which might suggest the breakdown of oral or even lung tissues that could be connected to pathological processes, including human hemoglobin (subunits alpha, beta, and delta) not present in the other studied calculus samples. In contrast, in sample NSZ120C we found abundant microbial taxa, and the early medieval sample ZH576C mostly contained various proteins from *Bos* sp., including milk proteins. These differences support the idea of high variability in the composition of the dental calculus between individuals (for examples of proteins, see Table 2; for proteins including identified peptides, see Table S6).

**Table 2:** Examples of Proteins Detected from Samples of Ancient Dental Calculus Samples (NSZ151C, NSZ120C, and ZH576C) Related to the Oral Microbiome and Diet^a^

protein (accession number)^b^	organisms	number of peptides (unique)
T-complex protein 1 subunit theta (G1SHZ8)^1^	*Oryctolagus cuniculus*	13 (13)
hemoglobin subunit beta (P68871)^1^	*Homo sapiens*	8 (4)
flagellar filament 33 kDa core protein (SEQF1598_00449)^2^	*Selenomonas sputigena*	12 (12)
flagellin (SEQF1674_00209)^2^	*Fretibacterium fastidiosum*	5 (5)
TonB-dependent receptor (SEQF2356_00667)^2^	*Porphyromonas gingivalis*	7 (7)
lactotransferrin (C7FE01)^3^	*Bos taurus*, *Bos indicus*	40 (3)
plasma serine protease inhibitor (Q9N2I2)^3^	*Bos taurus*	11 (7)
lactadherin (Q95114)^3^	*Bos taurus*, *Bos indicus*, *Bos mutus grunniens*, *Bos mutus*	8 (8)

a The organism hits are based on individual searches in Blastp non-redundant protein sequences.

b Unique to reported taxa within the set of identified proteins. ^1^—NSZ151C; ^2^—NSZ120C; ^3^—ZH576C.

Several proteins were assigned to oral bacteria such as *Selenomonas sputigena*, *Actinomyces dentalis*, and other *Actinomyces* spp., *Porphyromonas gingivalis*, or *Fretibacterium fastidiosum* (NSZ120C). We identified human proteins demonstrating an active immune reaction, such as complement C3 and C4 (NSZ120C) or hemoglobin (NSZ151C) that could result from bleeding caused by periodontal disease.

Dietary proteins belong to one of the most informative in the context of ancient dental calculus analysis, and thus they are often the primary focus of studies. Unfortunately, proteins related to milk consumption are frequent laboratory contaminants. However, in sample ZH576C, we found a large amount of bovine milk proteins (lactoperoxidase, lactotransferrin, and lactadherin) that were not present or were very minor in blanks. It suggests, together with other abundant proteins of *Bos* sp., consumption of milk and possibly other bovine products. Sample from NSZ 151 also contained other animal proteins that would imply contact or possibly consumption of, e.g., *Oryctolagus cuniculus* or *Sus scrofa*.

We also evaluated protein modifications as deamidation analysis was suggested to be a reliable tool for confirmation of the authenticity of ancient proteins and was used primarily for collagen, especially in case of very ancient samples that are thousands or even millions of years old.^26,53,54^ However, both sites studied here are only hundreds of years old and are well preserved, therefore deamidation levels of analyzed samples are far lower compared to, e.g., Pleistocene *Mylodon*.^26^ We include modifications of the reported peptides in Table S6. Deamidation is quite variable, it depends on many factors, and it has been proposed to be used rather as an indicator of protein preservation.^55,56^ We suggest, e.g., additional targeted DNA analysis of organisms of interest to confirm the presence of taxa identified by proteomic analyses, for example, by metabarcoding and targeted PCR as done by Sawafuji et al.^57^ This should be possible from a single piece of material, e.g., if performed by the unified protocol presented here, or as done by Fagernäs et al.^35^

### Confirmation of the Presence of Ancient DNA

To maximize the use of the sample material, we tested subsequent DNA extraction. The powder samples after protein extraction were treated with DNA lysis buffer, and the DNA content was analyzed.

The concentration of aDNA extracts from ancient tooth powder (ZH539T) is summarized in Table S7 and Figure S1. The Qubit dsDNA HS Assay Kit only measures double-stranded DNA; however, some of the DNA after SDT treatment will be single-stranded and therefore undetectable this way. The average yield after SDT incubation was 258.09 pg of DNA from 1 mg of powder without EDTA wash (sample group A), 199.15 pg/mg with EDTA wash (group B), and 411.35 pg/mg (group D) without SDT incubation, excluding samples with the lowest input of 1 mg. Thus, we can still recover about 63% of the aDNA from samples after protein extraction compared to the standard extraction of the aDNA from untreated samples. This is comparable to the 57% aDNA yield obtained using the unified decalcification protocol by Fagernäs et al.^35^ where the supernatant was used for DNA extraction and the pellet for protein extraction, i.e., in the opposite way to our approach. The DNA yield in our protocol is higher without the washing step between SDT and EDTA treatment, which was used to unify further analysis with samples without proteomic extraction (e.g., for metagenomics). Our protocol is time-efficient, with both extractions finished in 24 h compared to standard approaches of multiple days of demineralization, thus saving time, energy, and laboratory as well as personnel capacities.

The results of Sanger sequencing were consistent with previously determined haplogroup W4; however, samples with the lowest input amounts (4A–4D) produced very weak and damaged products that were harder to sequence with more apparent aDNA damage such as cytosine deamination (see Figure S2). Therefore, we suggest higher input when possible, even if aDNA analysis is feasible for input of about 1 mg of tooth powder, depending on the preservation. The amount needed for analysis might be lower for more concentrated materials such as dental calculus.

SNPs found in the whole mitogenome of ZH 539a are shown in Table S8, and the ancient DNA damage pattern is shown in Figure S3. The aDNA in extracts is identical; only yield and quality vary between the extraction groups.

Similar to Fagernäs et al.,^35^ a pellet of debris remains after protein and aDNA extractions, possibly allowing further analysis of microremains by light microscopy, potentially allowing three diverse analyses of a single precious material.

## Conclusions

This study describes a new method for parallel protein and DNA extraction that can be used on both recent and ancient teeth and dental calculus, enabling the most efficient use of very complex and precious material such as dental calculus. A significant advantage of this approach is that DNA and proteins are extracted from an identical piece of material, allowing for comparison of the obtained data and avoiding redundant sampling and material destruction as well as potentially allowing for further microscopic analysis of microremains. Our protocol is time-efficient: both aDNA and protein extractions can be performed within 24 h.

However, as the yield of DNA extraction in the combined protocol is about two-thirds of the yield of the standard protocol yield, it is not recommended for a purely genomically oriented experiment design.

The proteins identified in the samples were consistent with the expected results from teeth and dental calculus, including proteins of human, dietary, and microbiome origin, indicating the applicability of the procedure to ancient dental material.

## Supporting Information Available

Workflow description of protein extract processing, workflow description of LC–MS/MS with separate chapters for recent teeth and ancient material (tooth and calculus), detailed description of the data evaluation, including the databases used, amount of tooth powder from a recent sample that was used for extraction tests, amount of tooth powder from ZH539a or dental calculus used for testing of the protein extraction method on ancient material, custom database for screening the proteins of interest, average number of identified proteins achieved using different extraction buffers, number of identified proteins in different input weights of the pulverized ancient tooth, examples of proteins detected in samples of ancient dental calculus samples (NSZ151C, NSZ120C, and ZH576C) related to the oral microbiome and diet, concentration of aDNA extracts and final yield, and SNPs found in the whole mitogenome of ZH 539a (PDF)
Results of the quantification of DNA isolates using the fragment analyzer (PDF)
SNPs in the mitogenome of tested samples (ZH539T) after Sanger sequencing (PDF)
Analysis of the damage pattern (PDF)
Full results of proteomic analysis of recent tooth samples treated by different extraction buffers (XLSX)
Full results of proteomic analysis of ancient tooth samples of different weights (XLSX)
Full results of proteomic analysis of ancient calculus samples and blanks (XLSX)
