# Supplementary material for: Extraction Protocol for Parallel Analysis of Proteins and DNA from Ancient Teeth and Dental Calculus
Source: J Proteome Res. 2023 Sep 12;22(10):3311–9. doi: 10.1021/acs.jproteome.3c00370 (PMC10563166; doi:10.1021/acs.jproteome.3c00370)
Supplement: Supplementary file 1 — pr3c00370_si_001.pdf [file pr3c00370_si_001.pdf]

Supporting Information

Extraction Protocol for Parallel Analysis of Proteins and DNA from Ancient Teeth and Dental Calculus

Eva Chocholova[1], Pavel Roudnicky[2], David Potesil[2], Dana Fialova[1], Karolina Krystofova[2,3], Eva Drozdova[1], and Zbynek Zdrahal[2,3]

1 Laboratory of Biological and Molecular Anthropology, Department of Experimental Biology, Faculty of Science, Masaryk University, Kamenice 5, 62500 Brno, Czech Republic

2 Mendel Centre for Plant Genomics and Proteomics, Central European Institute of Technology, Masaryk University, Kamenice 5, 62500 Brno, Czech Republic

3 National Centre for Biomolecular Research, Faculty of Science, Masaryk University, Kamenice 5, 62500 Brno, Czech Republic

Table of Contents:

Supporting Material S1. Protein extracts processing. Workflow description.

Supporting Material S2. LC-MS/MS. Workflow description with separate chapters for recent teeth and ancient material (tooth and calculus).

Supporting Material S3. Data Evaluation. Detailed description of the data evaluation, including the databases used.

Table S1. Amount of tooth powder from a recent sample that was used for extraction tests.

Table S2. Amount of tooth powder from ZH539a or dental calculus used for testing of the protein extraction method on ancient material.

Table S3. Custom database for screening the proteins of interest.

Table S4. Average number of identified proteins achieved using different extraction buffers.

Table S5. Number of identified proteins in different input weights of the pulverised ancient tooth.

Table S6. Examples of proteins detected in samples of ancient dental calculus samples (NSZ151C, NSZ120C, ZH576C) related to the oral microbiome and diet.

Table S7. Concentration of aDNA extracts and final yield.

Figure S1. Results of the quantification of DNA isolates using Fragment Analyzer™.

Figure S2. SNPs in mitogenome of tested samples (ZH539T) after Sanger sequencing.

Table S8. SNPs found in the whole mitogenome of ZH 539a.

Figure S3. Analysis of damage pattern.

Separate files:

Datasheet 1 - datasheet1_extraction_buffers.xlsx Full results of proteomic analysis of recent tooth samples treated by different extraction buffers.

Datasheet 2 - datasheet2_weights.xlsx Full results of proteomic analysis of ancient tooth samples of different weights.

Datasheet 3 - datasheet3_ancient_calculus_blanks.xlsx Full results of proteomic analysis of ancient calculus samples and blanks.

Supporting Material S1. Protein extracts processing

Filter-aided sample preparation (FASP) was performed based on Wisniewski *et al*.[1]. Samples were loaded (7,000 x g) to 10kDa cut-off filter columns, washed (14,000 x g) with 8M urea buffer, alkylated with 50mM iodoacetamide (IAA), washed again first with urea buffer and then with 50mM ammonium bicarbonate (AB) buffer. Digestion was carried out overnight (18h) at 37 °C while shaking at 900 rpm, using 0.5 μg of trypsin (sequencing grade; Promega).

Protein input for FASP was in case of recent teeth ca 50 μg/sample (estimation based on bands density on 12% SDS-PAGE gel). In case of ancient tooth and dental calculus, we had to use whole extracts.

Supporting Material S2. LC-MS/MS

S2.1 Recent teeth analysis

LC-MS/MS analyses of all peptide mixtures were performed using the Ultimate 3000 RSLCnano system connected to the Orbitrap Fusion Lumos Tribrid mass spectrometer (Thermo Fisher Scientific). Prior to LC separation, tryptic digests (approx. 2 μg of peptides) were concentrated online and desalted using a trapping column (100 μm × 3 cm, 3.5 μm X-Bridge BEH 130 C18 sorbent, Waters; temperature 40 ºC). After washing the trapping column with 0.1% formic acid (FA), the peptides were eluted (flow rate - 300 nl/min) from the trapping column onto an analytical column (Acclaim Pepmap100 C18, 3 μm particles, 75 μm × 500 mm; at temperature 40 ºC, Thermo Fisher Scientific) using a 140 min linear gradient program (1-80% of mobile phase B; mobile phase A: 0.1% FA in water; mobile phase B: 0.1% FA in 80% ACN). Equilibration of the trapping column and analytical column was performed prior to injection of the sample into the sample loop. The analytical column outlet was directly connected to the Digital PicoView 550 (New Objective) ion source with the sheath gas option and the use of the SilicaTip emitter (New Objective; FS360-20-15-N-20-C12). ABIRD (Active Background Ion Reduction Device, ESI Source Solutions) was installed.

MS data was acquired using a data-dependent strategy with a survey scan (*m/z* range 350-2,000) selecting the top 20 most intense precursors. The resolution of the survey scan was 120,000 (at *m/z* 200) with a target value of 4×10^5 ions and a maximum injection time of 100 ms. HCD MS/MS (30% relative fragmentation energy, normal mass range) spectra were acquired with a target value of 5.0×10^4 and a resolution of 15,000 (at *m/z* 200). The maximum injection time for MS/MS was

22 ms. Dynamic exclusion was enabled for 30 s after one MS/MS spectra acquisition. The isolation window for MS/MS fragmentation was set to *m/z* width of 1.2.

S2.2 Ancient tooth and dental calculus analysis

LC-MS/MS analyses of all peptide mixtures of ancient samples were done using the nanoElute system connected to the timsTOF Pro mass spectrometer (Bruker). One column (separation column: Bruker, FIFTEEN, 75 µm ID, 150 mm long, 1.9 µm particles) mode was used on the nanoElute system with default equilibration conditions (4 column volumes at 800 bars). Sample loading was carried out using 2 pickup volumes + 2 µl at 800 bars. The peptides were eluted by a 90 min linear gradient program (flow rate 500 nl/min, 3-80% of mobile phase B; mobile phase A: 0.1% FA in water; mobile phase B: 0.1% FA in ACN). The column was placed inside the Column Toaster (40 °C; Bruker) and its emitter side was installed into CaptiveSpray ion source (Bruker). MS/MS data were acquired in the *m/z* range of 100-1700 and the $1/K_0$ range of 0.6-1.6 $V \times s \times cm^{-2}$ using the DDA-PASEF method acquiring 10 PASEF scans with a scheduled target intensity of 20,000 and an intensity threshold of 2,500. Active exclusion was set for 0.4 min with precursor reconsideration for 4× more intense precursors.

Supporting Material S3. Data Evaluation

From the obtained MS/MS spectra, the MGF files were generated using automated processing script in HyStar software (Bruker). MGF files were then analysed in the Proteome Discoverer software (Thermo Fisher Scientific; version 1.4) with in-house Mascot (Matrix Science; version 2.6.2) search engine utilisation. Data obtained from recent and ancient teeth were searched against protein databases of *Homo sapiens* (20,600 protein sequences, version from 2021-06-16, downloaded from http://ftp.uniprot.org/pub/databases/uniprot/current_release/knowledgebase/reference_proteomes/Eukaryota/UP000005640/UP000005640_9606.fasta.gz), Human Oral Microbiome (3,907,188 protein sequences, version from 20-01-09, downloaded from http://www.homd.org/ftp/genomes/PROKKA/current) and cRAP contaminants (112 sequences, version from 2018-11-22, downloaded from http://www.thegpm.org/crap). A custom database has been created for the searches of data from ancient calculus. It contained all databases mentioned above plus many other taxonomies, relevant to sample composition. The database content is in

Table S3. In addition, proteins identified during this search have been subsequently validated by searching against Uniref100 database (261,174,669 sequences, version from 2021-02-01 downloaded from http://ftp.ebi.ac.uk/pub/databases/uniprot/uniref/uniref100/uniref100.fasta.gz). The mass tolerance for the peptides and MS/MS fragments was 10 ppm and 0.05 Da, respectively. Modifications were set as follows for database search: oxidation (M, P) and deamidation (N, Q) as variable modifications, with carbamidomethylation (C) as a fixed modification. For the ancient calculus, oxidation on the P modification was not used. The enzyme specificity was tryptic with two permissible miscleavages. Rank 1 peptides with a minimum length of 6 amino acids and a Mascot expectation value < 0.01 were used for protein list generation. Proteins identified on at least two unique peptides are reported.

Proteins that were reported on the same or higher number of peptide spectrum matches in blanks and swabs of storage boxes than in ancient calculus samples were removed from the results.

All graphs were created in R (version 4.2.0) using ggplot2 (version 3.3.6) package[2,3].

Mass spectrometry proteomics data were deposited in the ProteomeXchange Consortium via PRIDE[4] partner repository under the dataset identifier PXD042668.

Table S1: **Amount of tooth powder from a recent sample that was used for extraction tests.**

Sample ID	Weight of tooth powder [mg]	Method of extraction
1	19.5	Citric acid 5% (w/v)
2	20.0	
3	19.6	
4	19.8	
5	19.5	Citric acid 10% (w/v)
6	20.0	
7	19.6	
8	19.7	
9	19.7	Hydrochloric acid 5% (v/v)
10	19.6	
11	19.9	
12	19.5	
13	20.0	Hydrochloric acid 10% (v/v)
14	20.0	
15	20.0	
16	20.0	
17	19.7	Sodium calcium edetate
18	19.9	
19	19.5	
20	20.0	
21	19.8	SDT buffer
22	19.8	
23	19.7	
24	19.6	

Table S2: **Amount of tooth powder from ZH539a or dental calculus used for testing of the protein extraction method on ancient material.**

Sample ID	Weight of material [mg]
1A	19.2
2A	9.4
3A	3.9
4A	0.9
NA (blank A)	-
1B	20.7
2B	10.3
3B	4.7
4B	1.0
NB (blank B)	-
1C	19.6
2C	9.4
3C	4.0
4C	0.9
NC (blank C)	-
5 (NSZ151C)	2.0
6 (NSZ120C)	23.6
7 (ZH576C)	11.4

Table S3: **Custom database for screening the proteins of interest.** The proteomic data were downloaded on 22.04.2022.

Taxonomy	Uniprot link/ID
Accipiter	taxonomy_8956
Allium	taxonomy_4678
Anatidae	taxonomy_8830
Apiaceae	taxonomy_4037
Apis	taxonomy_7459
Asteroideae	taxonomy_102804
Avena sativa	taxonomy_4498
Beta	taxonomy_3554
Brassica	taxonomy_3705
Camelina sativa	taxonomy_90675
Camelus	taxonomy_9836
Canidae	taxonomy_9608
Cannabis	taxonomy_3482
Cichorioideae	taxonomy_219120
Citrus	taxonomy_2706
Clupea harengus	taxonomy_7950
Coffea	reviewed_yes+taxonomy_13442 (reviewed)
Columbidae	taxonomy_8930
Corylus	taxonomy_13450
Cucurbitaceae	taxonomy_3650
Curcuma longa	taxonomy_136217
Cyprinus carpio	UP000694427
Ebola virus	taxonomy_1570291
Equidae	taxonomy_9788
Esox	taxonomy_8009
Falco	taxonomy_8952
Felis	taxonomy_9682

Galliformes	taxonomy_8976
Glycine	taxonomy_3846
Hordeum vulgare subsp. vulgare	UP000011116
Influenza A virus	reviewed_yes+taxonomy_11320 (reviewed)
Juglandaceae	taxonomy_16714
Juniperus	taxonomy_13100
Lamiaceae	taxonomy_4136
Lauraceae	taxonomy_3433
Lens	taxonomy_3863
Leporidae	taxonomy_9979
Linum	taxonomy_4005
Moraceae	taxonomy_3487
Musa	taxonomy_4640
Nicotiana	taxonomy_4085
Olea	taxonomy_4145
Oryza sativa	taxonomy_4530
Panicum miliaceum	UP000275267
Papaver	taxonomy_3468
Pecora	taxonomy_35500
Penaeus monodon	taxonomy_6687
Perca	taxonomy_8166
Phaseolus	taxonomy_3883
Phoenix dactylifera	taxonomy_42345
Pistacia vera	taxonomy_55513
Pisum sativum	pisum+sativum-filtered-organism__Pisum+sativum+(Garden+pea)+[3--
Plasmodium falciparum	reviewed_yes+taxonomy_5833 (reviewed)
Ribes	taxonomy_3801
Rickettsia prowazekii	UP000002480

Rosaceae	taxonomy_3745
Rotavirus A	reviewed_yes+taxonomy_28875 (reviewed)
Rumex acetosa	taxonomy_41241
Rutilus	taxonomy_48667
Saccharomyces cerevisiae	UP000002311
Saccharomyces pastorianus	taxonomy_27292
Sander	taxonomy_283033
Sardina	taxonomy_27696
Secale cereale	taxonomy_4550
Sesamum	taxonomy_4181
Setaria italica	UP000004995
Silurus	taxonomy_94992
Solanum	taxonomy_4107
Suidae	taxonomy_9821
Sus scrofa	UP000008227
Tanacetum vulgare	taxonomy_128002
Tinca	taxonomy_27716
Triticum aestivum	UP000019116
Triticum dicoccum	taxonomy_49225
Triticum turgidum	taxonomy_4571
Ursus arctos	taxonomy_9644
Urtica dioica	taxonomy_3501
Vaccinium	taxonomy_13749
Variola virus	taxonomy_ 10255
Vicia sativa	taxonomy_3908
Vitis	taxonomy_3603
Yersinia pestis	reviewed_yes+taxonomy_632
Zea	taxonomy_4575

Table S4: **Average number of identified proteins achieved using different extraction buffers.** The pooled powder from recent teeth was used here (REC1 and REC2). Only proteins identified with at least two unique peptides were considered.

Buffer	Proteins	Stdev	Origin
CA 5%	72.50	12.70	Only non-microbial
CA 10%	62.50	18.71	Only non-microbial
HCl 5%	86.33	4.78	Only non-microbial
HCl 10%	69.50	12.66	Only non-microbial
Edetate	302.25	34.26	Only non-microbial
SDT	608.00	91.97	Only non-microbial
CA 5%	74.00	14.54	Microbial and non-microbial
CA 10%	62.75	19.11	Microbial and non-microbial
HCl 5%	90.33	10.40	Microbial and non-microbial
HCl 10%	85.00	17.01	Microbial and non-microbial
Edetate	569.50	64.92	Microbial and non-microbial
SDT	1710.25	180.92	Microbial and non-microbial

Table S5: **Number of identified proteins in different input weights of the pulverised ancient tooth.** Only proteins identified with at least two unique peptides were considered. A, B, and C groups were used as replicates in proteomic analysis and were processed in the same way. Workflow of analyses of A, B and C is visualised in Figure 2: Samples of group A and B were analysed for both protein and DNA content, B had an added step of EDTA wash prior to DNA extraction. Sample group C was only analysed for proteomic content. Protein analyses were not influenced by the DNA extraction in any way, as they were done separately.

Input weight	Average number of proteins	Stdev	A group proteins	B group proteins	C group proteins
20 mg	8.00	1.73	9	9	6
10 mg	12.33	4.16	17	11	9
5 mg	6.67	1.53	5	8	7
1 mg	5.67	1.15	5	7	5

Table S6: **Examples of proteins detected in samples of ancient dental calculus samples (NSZ151C, NSZ120C, ZH576C) related to the oral microbiome and diet.** The organism hits are based on individual searches in Blastp non-redundant protein sequences. Only unique peptides from lactotransferrin are shown from the total of 40 peptides. PSMs = peptide-spectrum match. * unique to reported taxa within the set of identified proteins. [1] – NSZ151C; [2] – NSZ120C; [3] – ZH576C

Protein (accession number)	Organisms	Peptides + modifications	PSMs
CCT-theta (G1SHZ8)[1]	*Oryctolagus cuniculus*		
		EDGAISTLVIR*	2
		ETEGDVTSVKDAK*	1
		FAEAFEAIPR*	2
		GSTDNLMDDIER* + M7(Oxidation)	2
		HFSGLEEAVYR*	1
		ILGSGIYSSSVLHGMVFK*	2
		ILGSGIYSSSVLHGMVFKK*	1
		LATNAAVTVLR*	2
		LFVTNDAATILR*	4
		LVPGGGATEIELAK*	1
		NVGLDIEAEVPAVK*	6
		QITSYGETCPGLEQYAIK* + C9(Carbamidomethyl)	1
		XAVYSCPFDGMITETK* + C6(Carbamidomethyl); M11(Oxidation)	3
Haemoglobin subunit beta (P68871)[1]	*Homo sapiens*		
		EFTPPVQAAYQK*	6
		FFESFGDLSTPDAVMGNPK* + M15(Oxidation)	11
		GTFATLSELHCDK* + C11(Carbamidomethyl)	3
		LLVVYPWTZR	18
		VHLTPEEK	3
		VLGAFSDGLAHLDNLK	8
		VNVDEVGGEALGR*	10
		VVAGVANALAHK	9
Flagellar filament 33 kDa core protein (SEQF1598_00449)[2]	*Selenomonas sputigena*		
		ALDQQTTIGAVQSR*	15
		ANAAINVLDNAISK*	7
		AQDNSQDNSMNFQIGTR*	12
		DKANAAINVLDNAISK*	8
		ELNQSIDQINDNANITFNGK* + N18(Deamidated)	2
		INGAVDDASGYAISER* + N2(Deamidated)	9
		LNYTSQNLTTASENVQASESTIR*	5
		SANAVLDAFSETVR*	2
		SVGGITFSITDTQGNINK*	5

	VAEGAVSSTVDILK*	3
	VINAANDTNTDDDR*	2
	YLVDGTHNQK* + N8(Deamidated)	9
Flagellin (SEQF1674_00209)[2]	*Fretibacterium fastidiosum*	
	ELSVQSANDTLTQQDR*	2
	GYIQLEIDQLR*	1
	INSAADDAAGLAISEK*	9
	INSAADDAAGLAISEKMR*	1
	SITIIDNAIDKVSTQR*	1
TonB-dependent receptor (SEQF2356_00667)[2]	*Porphyromonas gingivalis*	
	AANGVIVITTK* + N3(Deamidated)	1
	AQAGFDFLQER*	1
	EAYQATMSGYAQLTPIK* + M7(Oxidation)	2
	ESTWLSDLR*	1
	LLENASFLR*	1
	LLPNNPLALDPLGTSR*	2
	VYGSATFNYNR*	1
Lactotransferrin (C7FE01)[3]	*Bos taurus, Bos indicus*	
	AAHVKQVLLHQQALFGK*	1
	QVLLHQQALFGK*	19
	QVLLHQQALFGKNGK* + N13(Deamidated)	2
Plasma serine protease inhibitor (Q9N2I2)[3]	*Bos taurus*	
	AQILEGLGIGPGEGSEEELHSASQR* + Q2(Deamidated)	8
	AVVEVDESGTQAAAATGMVITFK*	9
	DIFTSDADLTGISNHSSIR*	2
	EGEMEQVENGLK	2
	ELQQPQDSLQLSLGNALFTKPR*	1
	FSIEGSYQLEEVLPK	5
	LPIQEAFLGAMR*	4
	QQDQFYYLLDR	6
	STHEQDFYVTPETVVR	5
	TLYLADTFPTNFEDPEGAK*	2
	TLYLADTFPTNFEDPEGAKK*	1
Lactadherin (Q95114)[3]	*Bos taurus, Bos indicus, Bos mutus grunniens, Bos mutus*	
	DFGHIQYVAAYR*	1
	IFIGNVNNSGLK*	4
	INLFDTPLETQYVR*	5
	MWVTGVVTQGASR*	4
	QFQFIQVAGR*	1
	VAYGDDGVTWTEYK*	4
	VAYGDDGVTWTEYKDPGASESK*	2
	VTGLITQGAR*	2

Table S7: **Concentration of aDNA extracts and final yield.** The yield was calculated as pg of extracted DNA to mg of tooth powder or calculus used for extraction.

Sample number	Weight of tooth powder [mg]	DNA concentration [ng/µl]	Yield [pg of DNA to mg of powder]
1A	19.2	0.0846	264.38
2A	9.4	0.0418	266.81
3A	3.9	0.0158	243.08
4A	0.9	0.0130	866.67
NA	-	0	-
1B	20.7	0.0654	189.57
2B	10.3	0.0332	193.40
3B	4.7	0.0168	214.47
4B	1.0	0.0120	720.00
NB	-	0	-
1C	19.6	-	-
2C	9.4	-	-
3C	4.0	-	-
4C	0.9	-	-
NC	-	-	-
1D	22.9	0.129	338.00
2D	10.5	0.109	622.86
3D	4.7	0.0214	273.19
4D	1.1	0.0108	589.09
ND	-	0	-

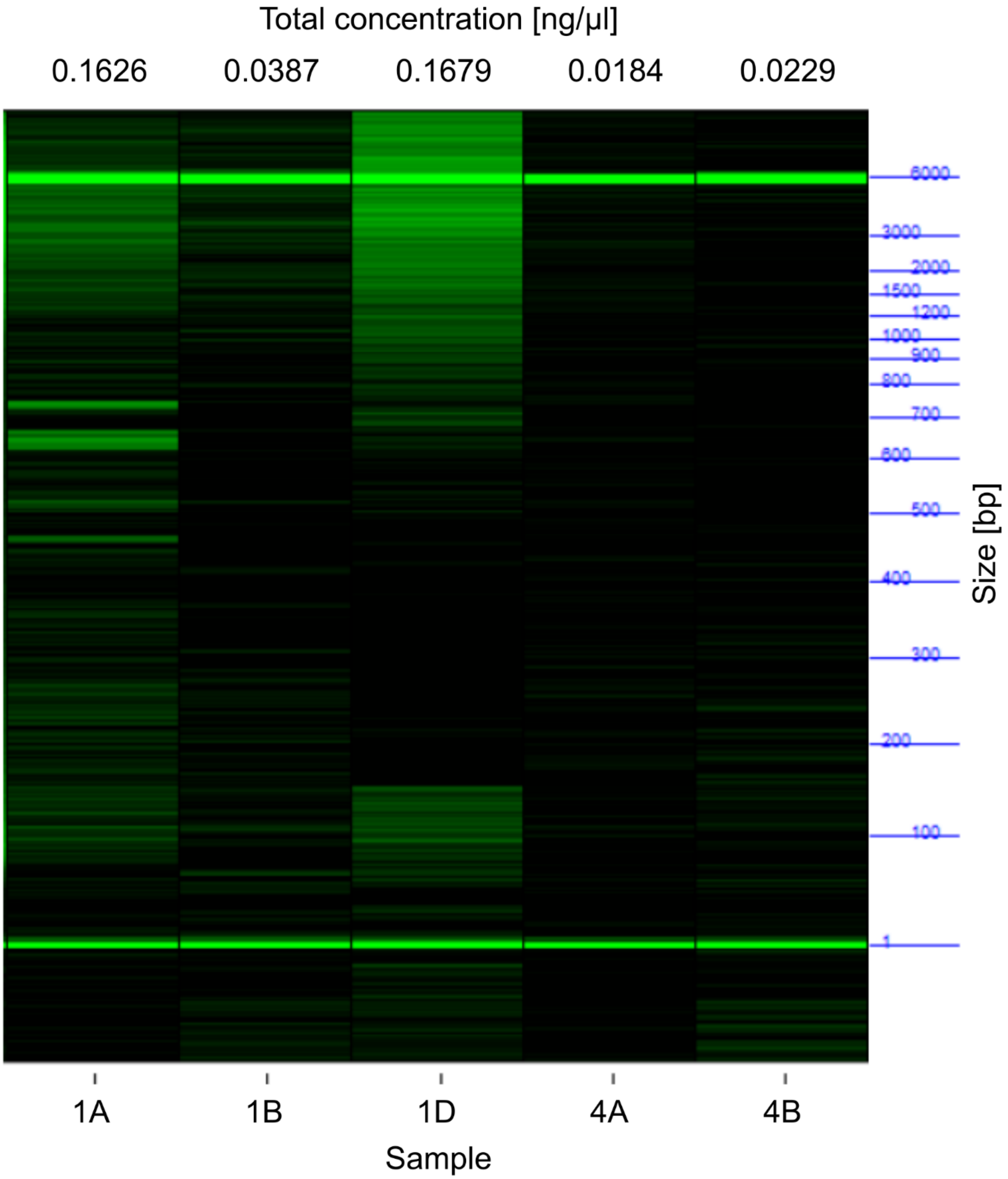

Fig. S1. Results of the quantification of DNA isolates. Quantification was performed on Fragment Analyzer™ using the DNF-474 High-Sensitivity NGS Fragment Analysis Kit (Advanced Analytical). Samples 1A, 1B, and 1D had 20 mg of input material, samples 4A and 4B had about 1 mg of input material, detection of sample 4D was not successful. The total concentration includes bacterial DNA that is a standard part of samples from archaeological

context and tends to have significantly longer fragments. No contamination with human DNA was detected based on Sanger sequencing of mitochondrial DNA.

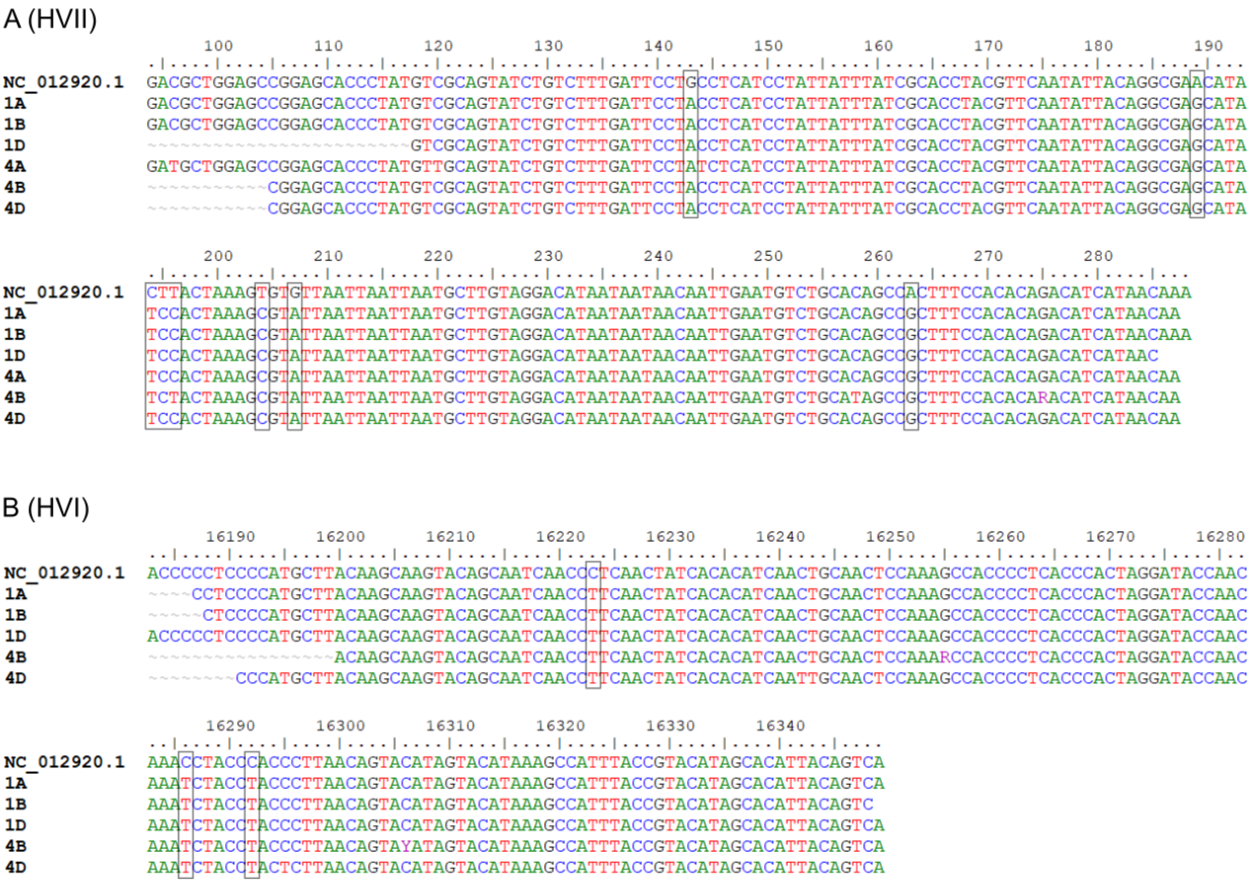

Fig. S2. SNPs in mitogenome of tested samples (ZH539T) after Sanger sequencing. A – HVII in range 94-288. B – HVI in range 16183-16349. Polymorphisms different from reference (NC_012920.1) are highlighted by grey frames and include G143A, A189G, C194T, T195C, T196C, T204C, G207A, A263G, C16223T, C16286T, C16292T. These SNPs are shared by most reads of the same sample and are considered authentic. Samples 4A-4D show a few nucleotide changes that are not standardly found in the W4 haplogroup and were only found in weak signal electrophoretograms. They are most probably a result cytosine deamination that is typical for ancient DNA (observable random changes C>T and G>A). Sample 4A failed to produce sequenceable products in HVI.

Table S8: **SNPs found in the whole mitogenome of ZH 539a.** Previously sequenced from tooth powder untreated by SDT. Underlined SNPs were found by Sanger sequencing of samples 1A-1D and 4A-4D in regions 94-288 and 16183-16349.

Predicted haplogroup	Number of variants	Variants
W4	39	A73G, G143A, A189G, C194T, T195C, T196C, T204C, G207A, A263G, T310C, G709A, A750G, T1243C, A1438G, G1503A, C1838T, A2706G, A3505G, A4769G, G5046A, G5460A, C7028T, G8251A, A8860G, G8994A, T9653C, C11674T, G11719A, A11947G, T12414C, C12705T, T14512C, C14766T, A15326G, G15884C, C16223T, C16286T, C16292T, T16519C

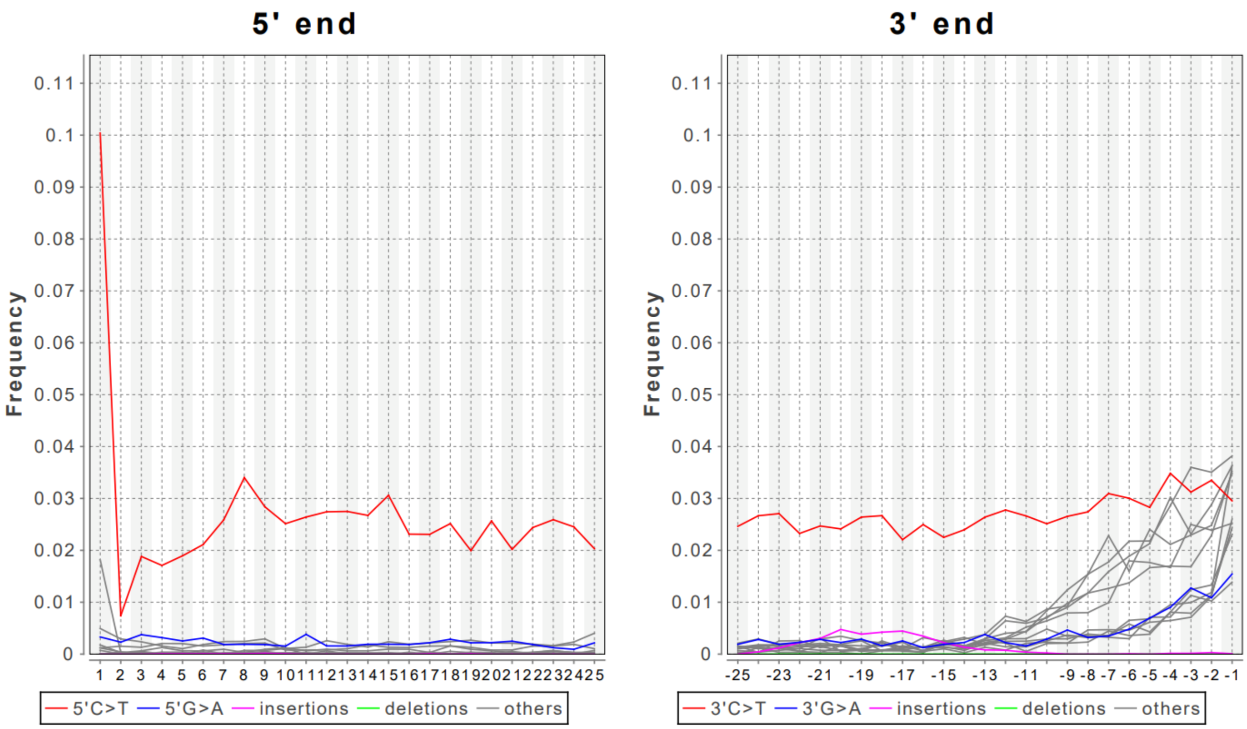

Fig. S3: Analysis of damage pattern. Performed by DamageProfiler in the whole mitogenome data of early medieval woman in burial ZH 539a.

References

(1) Wiśniewski J.R., Zougman A., Nagaraj N., Mann M., 2009. Universal sample preparation method for proteome analysis. Nat. Methods. 6, 359–362. https://doi.org/10.1038/nmeth.1322 PMID: 19377485

(2) R Core Team, 2022. R: A language and environment for statistical computing. R Foundation for Statistical Computing, Vienna, Austria. https://www.R-project.org/.

(3) Wickham H., 2016. ggplot2: Elegant Graphics for Data Analysis. Springer-Verlag New York. ISBN 978-3-319-24277-4, https://ggplot2.tidyverse.org.

(4) Perez-Riverol Y., Csordas A., Bai J., Bernal-Llinares M., Hewapathirana S., Kundu D.J., Inuganti A., Griss J., Mayer G., Eisenacher M., Pérez E., Uszkoreit J., Pfeuffer J., Sachsenberg T., Yilmaz S., Tiwary S., Cox J., Audain E., Walzer M., Jarnuczak A.F., Ternent T., Brazma A., Vizcaíno J.A., 2019. The PRIDE database and related tools and resources in 2019: improving support for quantification data. Nucleic Acids Res. 47(D1):D442-D450. 10.1093/nar/gky1106. PMID: 30395289; PMCID: PMC6323896.
