# Supplementary figures and images for: Extraction Protocol for Parallel Analysis of Proteins and DNA from Ancient Teeth and Dental Calculus
Source: J Proteome Res. 2023 Sep 12;22(10):3311–9. doi: 10.1021/acs.jproteome.3c00370 (PMC10563166; doi:10.1021/acs.jproteome.3c00370)

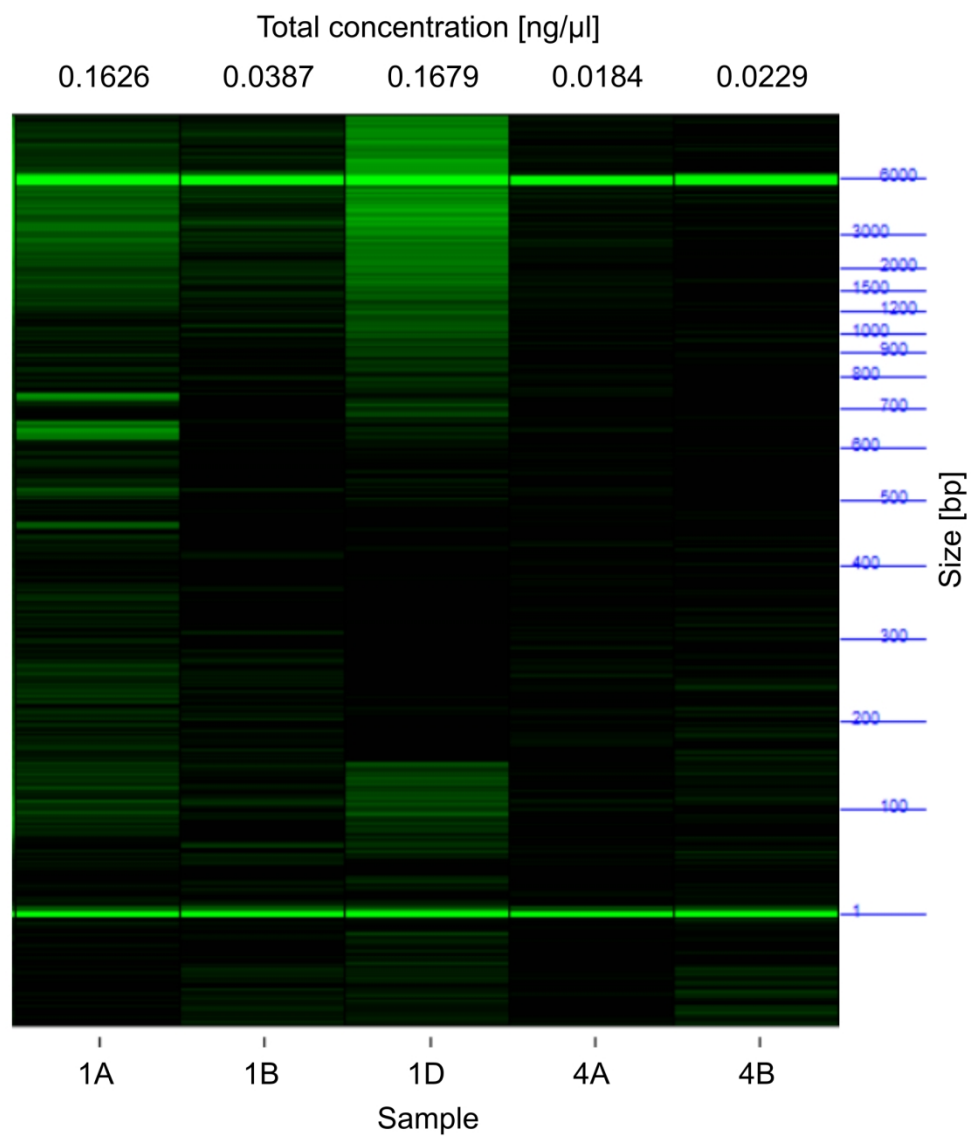

164x187mm (300 x 300 DPI)

Supplement: Supplementary file 2 — pr3c00370_si_002.pdf [file pr3c00370_si_002.pdf]

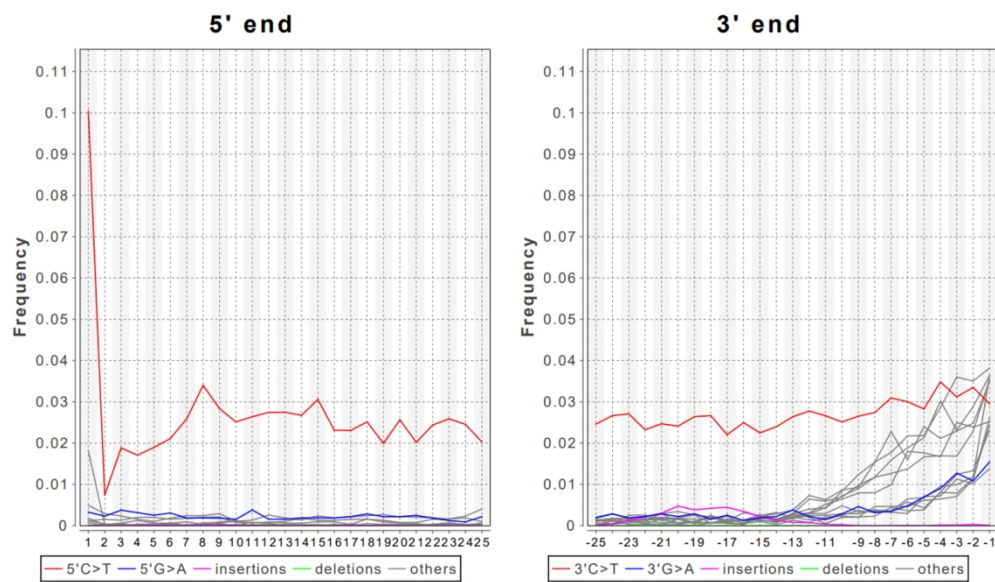

415x239mm (96 x 96 DPI)

Supplement: Supplementary file 4 — pr3c00370_si_004.pdf [file pr3c00370_si_004.pdf]
