# Supplementary material for: Extraction Protocol for Parallel Analysis of Proteins and DNA from Ancient Teeth and Dental Calculus
Source: J Proteome Res. 2023 Sep 12;22(10):3311–9. doi: 10.1021/acs.jproteome.3c00370 (PMC10563166; doi:10.1021/acs.jproteome.3c00370)
Supplement: Supplementary file 3 — pr3c00370_si_003.pdf [file pr3c00370_si_003.pdf]

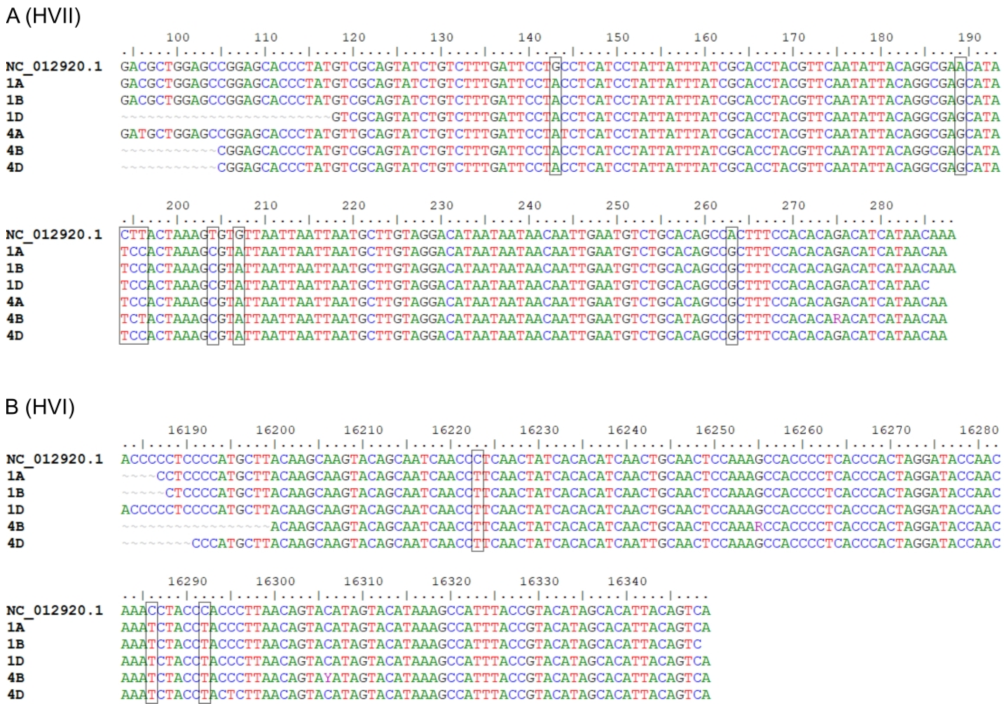

330x230mm (300 x 300 DPI)
